# Challenges of and possible solutions for living with endometriosis: a qualitative study

**DOI:** 10.1186/s12905-022-01603-6

**Published:** 2022-01-26

**Authors:** Gabriella Márki, Dorottya Vásárhelyi, Adrien Rigó, Zsuzsa Kaló, Nándor Ács, Attila Bokor

**Affiliations:** 1 Doctoral School of Psychology, Eötvös Loránd University, Budapest, 1064 Hungary; 2 Institute of Psychology, Eötvös Loránd University, Izabella Street 46, Budapest, 1064 Hungary; 3 Department of Obstetrics and Gynecology, Faculty of Medicine, Semmelweis University, Baross Street 27, Budapest, 1088 Hungary

**Keywords:** Endometriosis, Health-related quality of life, Psychosocial impact, Focus group, Coping

## Abstract

**Background:**

Endometriosis as a chronic gynecological disease has several negative effects on women’s life, thereby placing a huge burden on the patients and the health system. The negative impact of living with endometriosis (impaired quality of life, diverse medical experiences) is detailed in the literature, however, we know less about patients’ self-management, social support, the meaning of life with a chronic disease, and the needs of patients. To implement a proper multidisciplinary approach in practice, we need to have a comprehensive view of the complexity of endometriosis patients’ life and disease history.

**Methods:**

Four focus group discussions were conducted between October 2014 and November 2015 by a team consisting of medical and psychological specialists. 21 women (age: 31.57; SD = 4.45) with surgical and histological confirmation of endometriosis were included in the study. Discussions were audiotaped and transcribed verbatim, and a 62,051-word corpus was analyzed using content analysis.

**Results:**

Four main themes emerged from the analysis: (1) the impact of endometriosis on quality of life, (2) medical experiences, (3) complementary and alternative treatments, and (4) different coping strategies in disease management. All themes were interrelated and highly affected by a lack of information and uncertainty caused by endometriosis. A supporting doctor-patient relationship, active coping, and social support were identified as advantages over difficulties. Finding the positive meaning of life after accepting endometriosis increased the possibility of posttraumatic growth. Furthermore, women’s needs were identified at all levels of the ecological approach to health promotion.

**Conclusions:**

Our results highlight the need for multidisciplinary healthcare programs and interventions to find solutions to the difficulties of women with endometriosis. To achieve this goal, a collaboration of professionals, psychologists, and support organizations is needed in the near future.

## Introduction

Endometriosis is a chronic inflammatory disease that is defined as the presence of endometrium-like tissues outside the uterus causing pain symptoms (dysmenorrhea, dyspareunia, chronic pelvic pain) and infertility [1]. This gynecological disease affects approximately 2–10% of the reproductive-aged and 50% of infertile women, and women with endometriosis have increased risk of obstetric outcome [2]. Because symptoms are not specific, the diagnostic delay is almost 8–10 years [3].

Endometriosis has a negative impact on health-related quality of life (HRQoL) [4]. Quantitative studies identified deterioration in physical wellbeing [5], psychological functioning [6, 7], daily life activities and work productivity [8, 9], social participation [10], quality of sexual life [11], and an increase in financial burden [12]. Decreased HRQoL has a negative feedback effect on endometriosis progression [13, 14]. Furthermore, it is already known that pain is a major cause of these physical, psychosocial, emotional, and work-related difficulties among patients [15–17].

Previously published qualitative data demonstrated the negative impact of endometriosis on HRQoL and medical experiences but offered fewer findings of self-management, social support, femininity, the meaning of life with a chronic disease, and future directions and needs of patients. Therefore, this study aims to expand knowledge of (i) the difficulties women have when living with endometriosis and (ii) their opportunities and mechanisms for coping with the negative impact of the disease. We assume that by exploring these main areas we can help to develop health promotion strategies to reduce further negative effects on women's lives.

## Methods

### Study design, procedure, and data collection

This qualitative study is part of a comprehensive study on the psychosocial aspects of endometriosis conducted by Eötvös Loránd University in conjunction with Semmelweis University, Budapest, Hungary. Participants with surgical and histological confirmation of endometriosis from our previous study [18] were invited via e-mail to participate in exploratory focus group discussions, where participation was voluntary. The invitation explained the nature and details of the study. Four focus group discussions were conducted in the department room of the Institute of Psychology, Eötvös Loránd University between October 2014 and November 2015. Within the postpositivist qualitative paradigm [19] we followed the phenomenological approach to inquiry. Focus groups allow data to be collected through a group, where participants express their opinion more naturally and influence one another, thus it is more likely that new issues will be raised than in a one-to-one interview. Furthermore, focus groups allow the perceptions, emotions, and concerns of participants to be explored [20, 21].

Focus group participants were asked to articulate their concerns and experiences on two topics: (i) living with endometriosis, (ii) disease-management, and experiences of medical or other treatments. All conversations were guided by the first author and trained assistant, who took field notes. Focus groups were audiotaped with their permission and transcribed verbatim by the assistant. Overall, there were 462 min of recording which were then transcribed into a 62,051-word corpus for the analysis.

### Analysis

The verbatim transcripts included typical or relevant non-verbal expressions (laughing, long pauses) that were confirmed by the assistant who made observations during focus group sessions. The basic element of analysis was the word. After checking the transcript (rereading the text while listening to the voice recording), the text was analyzed line by line using content analysis [22, 23] in ATLAS.ti by two independent coders [24]. They discussed and compared collected codes from the data and after reaching consensus code groups, defined categories and created themes. Final themes and categories were checked against codes [22].

## Results

### Sample characteristics

The study involved 21 patients diagnosed with endometriosis with a mean age of 31.57 (SD = 4.45). Participants (> 18 years) represented a homogenous group in terms of socio-economic background and ethnicity. On average, participants saw more than three gynecologists (range 1–15) and one alternative healer (range 0–4) before being diagnosed with endometriosis. The diagnostic delay was 2.05 years (SD = 3.32; range 0–12). Most participants (66.7%) had endometriosis-related symptoms by the time of the study. In all our participants, peritoneal endometriosis was observed (21/21, 100%), ovarian endometriosis was found in 11 patients (11/21, 52.3%) while deep infiltrating endometriosis affecting the rectum and/or the rectovaginal space was present in 6 cases (6/21, 28.6%). None of our patients had extrapelvic or abdominal wall endometriosis. Most patients of the whole sample used medical hormonal treatment at the time of the conversation. From the whole cohort, 18 (18/21, 85.7%) women received combined oral contraceptive therapy on a continuous regimen. We have observed no difference between the types of oral contraceptives since they were all combined pills containing dienogest and ethinylestradiol. Most of our participants (16/21) struggled to get pregnant, only one of them had a successful clinical pregnancy and delivery at the time of the conversation, further 23.8% of participants were undergoing in vitro fertilization (IVF). None of study participants had a comorbid psychiatric disorder.

### Thematic analysis findings

**Fig. 1:**
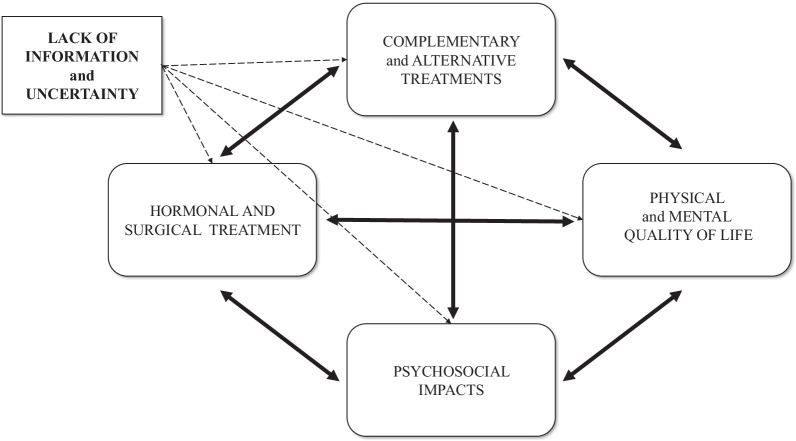
A model of the dynamic relationship of endometriosis-related themes and negative impacts

Four main themes emerged from the analysis: (a) impact of endometriosis on quality of life, (b) medical experiences, (c) complementary and alternative treatments, (d) different coping strategies in disease-management (Table 1). Notably, that all themes were highly affected by a lack of information and uncertainty related to endometriosis, while all the emerged themes showed a dynamic connection between them and present the patients’ circular pathways (Fig. 1).

**Table 1:** Themes, categories and subcategories that emerged from the data

Themes	Categories	Subcategories
**Impact on quality of life**		
	Physical impacts	
	Psychological impacts	
	Psychosocial impacts	*Social and family life*
		*Intimate relationship*
		*Sexual life*
		*Fertility*
		*Femininity*
		*Employment and education*
		*Financial impact*
**Medical experiences**		
	Diagnostic delay	*Normalization of symptoms*
		*Doctor delay*
	Treatment of endometriosis	*Pharmacological treatments*
		*Surgery and surgical experiences*
		*Childbearing as a treatment option*
		*ART treatment*
		*Recurrence of endometriosis*
	Doctor-patient relationship	
**Complementary and alternative treatment**		
	Lifestyle changes as treatment	*Diet and nutrition*
		*Physical activity*
	Psychology	
	Naturopathy and other methods	
**Different coping strategies in disease-management**		
	Obtaining information	*Information provided by doctors*
		*Internet*
		*Fellow patients*
	Active control and emotion-focused coping	*Self-care*
		*Positive attitude*
	Social support	*Personal relationships*
		*Endometriosis community*

### Theme 1: Impact of endometriosis on quality of life

#### Physical impacts

Most women mentioned chronic pelvic pain, dysmenorrhea, and dyspareunia as leading symptoms of their endometriosis. Most of them reported that pain killers or special body positions did not significantly relieve pain.

It was never-ending, so I lived like this every day. I kneeled on the ground. I moved back and forth because it was not good any other way and yet I still held tightly onto my hair because I was in so much pain.

#### Psychological impacts

Besides the physical burden, participants also reported psychological consequences of endometriosis, namely *anxiety, stress, and helplessness*, and sometimes these were more confusing and annoying than the physical symptoms. One participant described how depressing it was to realize that she had lost 10 years of her life living in permanent pain without receiving the correct diagnosis and treatment. Feelings of *loss and shame* were also highlighted by participants. *Uncertainty* about the possible recurrence of the disease has been identified as a further *stress* factor in women who wanted to take an active role in their disease management. The negative emotional state negatively shaped their way of thinking, and subsumed their everyday lives.

You can’t do anything about the physical side anymore, but the psychological aspects, they leave their mark on you. I gave in to endometriosis, in fact, my whole life revolved around it. It made me bitter, and I realized after a while that I couldn’t think about anything else.

#### The psychosocial effects of endometriosis

This category includes common and cumulative effects of physical and psychological impacts. Endometriosis-related uncertainty had several negative impacts on women’s life. *Families and friendships* were affected by a lack of adequate information and a feeling of helplessness.

Friendships were ruined at that time. There was not one aspect of my life that was not affected by endometriosis. Within the family, you can release the stress that you cannot release anywhere else. It was common for me to cry during family dinners. Even friends who were supportive did not always understand what I was going through.

*Intimate relationships* were negatively affected by uncertainties. Participants mentioned that explaining the disease and giving reassurance to their husbands was difficult. Women agreed that a supportive partner can be the biggest source of help and support, but not every relationship was able to handle the burden of endometriosis.

It [endometriosis] cost me my marriage… At that time, we had already started in vitro fertilization. The first one ended up in colonic obstruction and I got a stoma for three months. Before the next round of IVF started my ex-husband said it was over for him.

Some women experienced *sexual problems* and the inconvenience of sharing their experiences of dyspareunia due to the normalizing reaction of society and health care providers. The non-sharable experiences led in two cases to sexual aversion, when “*sex was equal to pain”.*

Besides dyspareunia and sexual dysfunctions, the most burden for most women were *fertility problems.* Participants stated that they pursued one of two options: some women insisted on childbearing and did not give up even after the defeats and inconveniences of IVF, because they thought it was worth the sacrifice; others re-evaluated pregnancy and went on to consider other options for motherhood.

We try and we hope, and I don’t know. I have learned a big lesson from this—that it would be nice to have a baby, but what if I can never have my own baby—because it could happen. Now I can say it out loud: it is okay, I can adopt a child or choose other options.

*Female identity* was negatively affected by infertility, sexual problems, and impersonal medical examinations. Repression and negative attitudes towards femininity have been mentioned as possible causes of their disease.

If you do not experience your femininity, it will come back to haunt you at some point.

Endometriosis and its treatment had a significant impact on participation in *education and employment*. Women mentioned sick-leave and semester deferral due to dysmenorrhea, as well as sleeping problems and surgery. The impact on employment usually depended on the boss and the flexibility of the workplace.

It can cause a lot of tension, finding where the line is between asking your boss to let you leave and be patient, or feeling that you are risking your job and tomorrow maybe you don’t have to go to work anymore.

The cost of gynecological consultations, medications, surgery, healthy nutrition, and further treatments caused a *financial burden* and required a considerable amount of time and energy*.*

The costs associated with endometriosis are so high; my family has an emergency budget just for this.

### Theme 2: Medical experiences

#### Diagnostic delay

Participants usually experienced that health professionals normalized symptoms of dysmenorrhea. It was not only normalization but physicians’ lack of adequate knowledge relating to endometriosis that caused misdiagnosis and diagnostic delay.

I went from doctor to doctor for seven years and I knew something was wrong because I could not conceive, so we were looking for the reason behind it. But a lot of doctors did not recognize the disease and that was the biggest problem.

#### Treatment of endometriosis

The option of *pharmacological treatments* was divisive among participants; most of them were concerned about side effects. Participants reported being fearful before *surgery* and stated that they were concerned about reproductive organs and intestinal involvement or getting stoma. Fear and uncertainty were pronounced concerning recovery and lack of information right after surgery. Participants reported that *having a child* was usually expressly recommended by gynecologists as a potential treatment option***.*** These women often experienced medical and social pressure to have a baby, even if they did not feel ready to become mothers.

A woman can find herself in this trap. Although the gynecologist means well, saying you must have a child as soon as possible is such a burden on the woman. It is unbearable and impossible to process.

*Infertility* was a sensitive topic in every discussion. Participants who underwent assisted reproductive technology treatment described it as impersonal, physically, and mentally stressful, for men as well. Furthermore, the possible *recurrence of endometriosis* proved to be one of the biggest uncertainty factors, and it placed a huge burden on women.

#### Doctor-patient relationship

Most women agreed that having a good, reliable gynecologist specialized in endometriosis is one of the most essential factors in managing endometriosis. Many participants had negative experiences with doctors who were negligent or had insufficient *professional knowledge* of endometriosis, which increased *diagnostic delay* by several years. Women highlighted that healthcare professionals’ uncertainty led to mistrust, increased fear, and despondency, and caused them to go ‘doctor-shopping’ because they could not accept their doctor’s negligent attitude towards their symptoms or recommended *treatment* options. All the women agreed that physicians who reassured and informed them properly as a specialized professional in endometriosis engendered the most trust.

It was an odd experience, that even doctors can’t tell me what is wrong with me and what will make me feel better. So, you have to go until you find someone you can at least trust.

### Theme 3: Complementary and alternative treatments

This theme includes women’s motivation towards all kinds of complementary and alternative treatments which may supplement or substitute medical treatments.

#### Lifestyle changes as treatment

Despite a lack of scientific evidence and findings of the positive effects of *lifestyle change*, women wanted to achieve better physical health and HRQoL and long-term recovery.

You have to be very conscious and responsible and need an incredible amount of time to develop this routine. I was exhausted and I wanted nothing more than to go to bed, but I knew if I did not prep my lunch for the next day, then I wasn’t going to have a [healthy] meal.

These women were given a great deal of contradictory information about their potential endometriosis *diet*. Those following a strict diet said it was like being a prisoner and they suffered because of the financial cost. When the diet was ineffective or too strict, women gave in and started to follow the needs of their bodies and developed a unique, personalized diet.

When I accidentally ate something, which was forbidden in the diet I would hate myself. Now I listen to my body, the things it likes or does not like.

Although it is difficult to find enough time and mobilize resources, all women agreed that *physical activity* is an essential part of managing the disease. Women were doing various sports (yoga, running, cycling, Zumba, swimming, intimate muscle training, and Pilates) regularly, but the efficacy of these sports was not specified during discussions.

#### Psychology

Due to the unknown etiology of the disease, participants stated that they had thought about the psychosomatic, stress-related origin of endometriosis. Many women sought psychological help by using cognitive methods, schema therapy, EMDR (Eye Movement Desensitization and Reprocessing), stress management, autogenic training, meditation, and hypnosis to alleviate their symptoms.

I went to a psychologist, and I opened up about this stuff [dyspareunia]. She pointed out things that I could not see myself, and for some reason, I believe that if I defeat this misery the endometriosis will disappear, too.

#### Naturopathy and other methods

A wide range of naturopathic medicine (acupuncture, reflexology, Chinese medicine, Ayurveda, kinesiology, herbs) was mentioned in focus group discussions. When women find no answer in western medicine, they seek help through alternative treatments.

Then I decided to start taking a path which I normally would not take, as the path that I am currently on is not working.

Participants were not able to agree about the impact of the aforementioned methods, because each woman had a different view of those effects.

### Theme 4: Different coping strategies in disease-management

#### Obtaining information

**Fig. 2:**
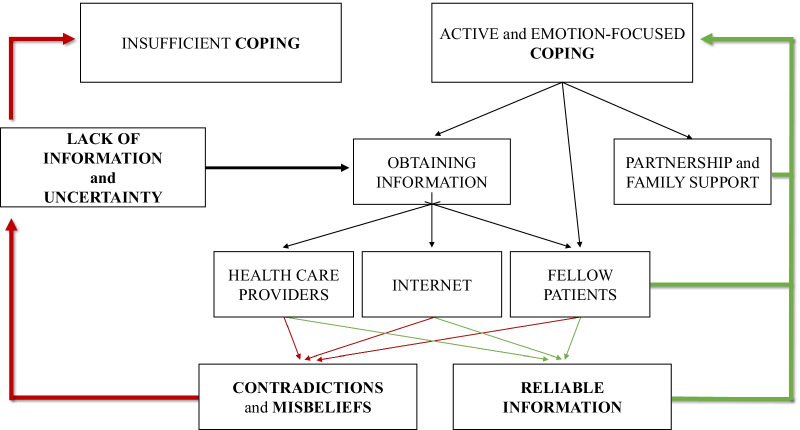
The model of sufficient and insufficient ways of coping with endometriosis

One of the most important aspects of disease-management was obtaining reliable information. Women were motivated to access as much information as possible, however, it was the area with the most obstruction. Contradictory information increased the feeling of uncertainty (see Fig. 2). *Insufficient information* from health care professionals also increased *uncertainty*. Only some women felt that they were properly informed by their gynecologists, and many of them found that they had to drag the information out of their doctors. After the diagnosis, some participants were sent home to read about endometriosis on the *internet*.

When you are sent home to look into it on the internet, it’s like being thrown into the sea in order to teach you how to swim.

All women experienced that the internet is *full of contradictions* and *misconceptions*. Furthermore, destructive opinions, negative experiences, and rumors from fellow patients on blogs and online forums often *confused* them. Some women learned the most about endometriosis from *fellow patients* in the waiting room, where they were able to exchange experiences and inform one another. However, they also drew attention to the distress they had experienced.

We [fellow patients] can understand each other’s problems because we are in the same boat, but if I get no positive feedback and I can’t say anything to her that she needs, it’s not good.

#### Active control and emotion-focused coping

Women described a wide range of active and emotion-focused coping mechanisms they need to be able to use flexibly. In addition to obtaining information, most women avoided passivity and took control, assuming an active role and *self-care* in managing endometriosis. Some women stated that since changing their lifestyle, they have been able to live a full life. Others mentioned the importance of listening to the signs and needs of their bodies. Many participants coped with the difficulties and uncertainty of endometriosis by having a *positive attitude*, trying to find the positive aspects, and trying to remain optimistic.

Endometriosis taught me to take care of myself, and try to heal myself, to listen to my body and my inner voice, to look for methods that might help, and to find those which really help.

#### Social support

All women agreed on the importance of *social support and support from their partners*. *“I cannot tell you how much it helps when he* [male partner] *stands by you.”* They were able to cope with living with endometriosis, operations, and treatment thanks to the personal support of *relatives and friends*. Several participants mentioned the important role played by *endometriosis community* members, who can give support by sharing intimate experiences of endometriosis so that women do not have to face their problems alone.

It is always nice to get support from others who have experienced similar things and similar problems, so it feels good to talk about it. You are not alone.

## Positive meaning of life after accepting endometriosis

Women described the difficulties, uncertainties, and lack of information surrounding endometriosis as pervasive features of their lives. Nevertheless, despite many difficulties and problems, women described a positive impact (*peace, patience, openness, personality development, and gratitude*) on their life after accepting their condition.

A whole new world has opened up before me. I am not saying that it is good to have endometriosis, but I have completely changed because of it. I would not be the same person if I had not gone through this. I improved as a person, and the journey is not over yet. I would not be as open towards people, I would not have these kinds of relationships, my family and my relationship would not be the same. I have a sense of purpose.

## Possible responses to “do patients know what they need?”

Focus group discussions allowed women with endometriosis to demonstrate their desire to take an active role in the management of their disease and to express their needs and options for alleviating the difficulties and deficiencies. These suggestions allow us to understand the real needs of women with endometriosis and design a proper health promotion program.

1. Giving *proper information from reliable sources* could be one of the best ways of reducing uncertainty and increasing HRQoL. Participants highlighted the need for information about surgical results right after the *postoperative* wake-up, which would reduce postoperative stress, anxiety, and uncertainty.
2. Women suggested that diagnostic delay, the risk of misdiagnosis, and the normalization of dysmenorrhea could be reduced through more extensive training and by *improving the specialist knowledge* of a broader range of health care professionals and medical students in all related medical areas in relation to the recognition of endometriosis.
3. Almost every woman agreed that clinical or health *psychologists* are needed in hospitals to help cope with diagnosis and surgery and to process disease-management.
4. All women agreed on the importance of *raising awareness* of endometriosis by *involving male partners, friends, and colleagues.* Educating and informing men about endometriosis would have long-term advantages, as men could provide effective help and support to women with endometriosis. Women highlighted the fact that it could also be very stressful for men to be involved, and that because of many uncertainty factors there should be educational and supportive groups for men as well.
5. To prevent more severe conditions, participants agreed that awareness of and education about endometriosis is necessary from menarche. *Preventive and educational programs* relating to endometriosis in schools would help ensure the early diagnosis of future patients.
6. Furthermore, women stated that it is a social responsibility to increase *publicity and awareness* of endometriosis in society, and a campaign like that for breast cancer would help to educate all social groups.

## Discussion

In our study, we first report a mutual dynamic connection between the main endometriosis-related themes (HRQoL, medical experiences, complementary and alternative treatment, and coping strategies), and show that these areas are negatively influenced by the most prominent themes: uncertainty and lack of information. Exploring the connections between these themes will also help to understand patient pathways, which is essential for planning the long-term management of women with endometriosis.

Identified topics are comparable with previous findings [25, 26], where negative impact on HRQoL and medical experience of endometriosis appeared as essential topics. Our results highlight that these themes are not independent of one another (see Figs. 1, 2). Prolonged (pain)symptoms of endometriosis decrease quality of life, and direct women to health care, where patients can face a variety of different experiences. An inadequate doctor-patient relationship affects not only medical experiences and the physical condition of patients but also impairs adherence, compliance, and HRQoL. Ineffective medical attention or treatment affects women’s relationship with healthcare and leads them to use (non)evidence-based alternative treatments. Patients need active, emotion-focused coping strategies which are properly supported by positive medical experiences, reliable information, and effective social support. In their absence, patients may use inadequate coping options, which can have a negative impact on HRQoL. Lifestyle change as a potential coping and disease-management strategy [27] is an obvious opportunity for women to have control over one aspect of their condition. Nonetheless, the effectiveness of nonmedical treatments in endometriosis has not been sufficiently explored by evidence-based medicine [3]. Our results highlight the importance of finding a scientific response to women’s questions because failed attempts have a negative impact on prognosis, quality of life, and self-esteem [25].

Uncertainty and lack of information can have a direct impact on HRQoL, medical experiences, coping, and indirectly, on fertility as well [15]. The normalization and rejection of symptoms as a general problem impact the doctor-patient relationship before diagnosis and leads to diagnostic delay and eliminates the benefits of early diagnosis [3].

The lack of information at health care centers causes women to seek self-management strategies [15, 28, 29]. The lack of information causes women to seek self-management strategies. Women try to obtain information from various sources, but they come across a great deal of contradictory information, which needs to be dealt with. Studies identified that becoming assertive and taking control can be a potential coping mechanism before diagnosis and treatment [28], but there are fewer findings of how women cope with endometriosis and achieve an asymptomatic and fertile life after diagnosis. The women in our study used positive emotion-focused coping strategies to focus on the positive and optimistic aspects of their lives. Besides, problem-focused coping (versus non-adaptive focus on emotions) was found as an adaptive and assertive coping strategy that correlates with lower stress and depressive symptoms. [30]. On the other hand, catastrophizing is a negative cognitive and emotional coping response to pain [31] and enhances pain perception as a predictor among women with endometriosis [32]. Roomaney and Kagee [33] highlight—in line with our results—that both problem-focused and positive emotion-focused coping strategies can be helpful for women with endometriosis. A third means of coping is based on the help and support provided by personal relationships and endometriosis communities. Strong relationships were characterized by admiration for women’s courage, independence, and inner strength [34, 35]. Self-help groups and endometriosis foundations can provide effective support to women from the individual (see reliable information; health promotion programs) [36] to society (see social awareness and publicity) [37, 38].

In addition to negative consequences and needs, there were some interesting findings supporting the results of Facchin et al. [22] about finding the meaning of life with endometriosis. Women with positive emotion-focused coping strategies and a lower level of stress can accept the disease and find positive meaning in their lives from endometriosis. These results suggest the possibility of posttraumatic growth (PTG) in endometriosis. PTG is defined as the “positive psychological change experienced as a result of the struggle with highly challenging life circumstances” [39] (e.g. chronic disease as trauma or danger to health). Previous studies on women with chronic disease identified that PTG is negatively associated with age, depression, and stress, while positively associated with time since diagnosis, education, income, social support, mental HRQoL, self-efficacy, self-esteem, and optimism [40–44]. These characteristics show similarities with predictors of mental health quality in endometriosis [18, 45], although to the author’s knowledge PTG in endometriosis patients has not been measured yet. The authors suggest that PTG may occur due to multiple health behavior changes which improve active coping and the patient’s sense of control [46–48]. Therefore, it is recommended that the possibility of PTG be explored in future endometriosis studies.

The authors acknowledge that are some limitations to the current study. Firstly, the study sample was low and consisted of participants with homogeneous demographic and disease characteristics. Secondly, we collected our data retrospectively. We asked women about their experiences about living with endometriosis without making differences in the pre- and post-operative period, because we wanted to collect all the affected areas in their life. Although there can be differences before and after the endometriosis surgery for example in the quality of sexual life [49]. These differences can be analyzed in further qualitative studies.

Thirdly, as endometriosis is a benign disorder, the primary objective of any treatment should be to alleviate symptoms, control progression, and improve quality of life. Laparoscopic surgery is the most widely accepted surgical approach in cases of peritoneal, ovarian, and deep infiltrating endometriosis (DIE) [3, 50, 51]. Peritoneal disease can be excised or vaporized using different energy sources, while ovarian endometriosis can be managed by cystectomy or ablation. According to the recent data the ovarian cystectomy may lead to the loss ovarian reserve [52]. The optimal type of colorectal resection in case of bowel DIE, whether conservative (shaving, disc resection) or radical technique (segmental bowel resection) has to be applied is under discussion [53–57]. It has been suggested that the conservative surgical therapy of colorectal DIE is associated with lower morbidity, however the unequivocal evidence supporting this hypothesis is still lacking. The external validity of present data regarding the surgical therapy of endometriosis should be investigated in future multicentric prospective randomized trials on a large cohort of patients. A clear limitation of our study is that we did not assess the impact of different surgical methods on the endometriosis related quality of life in our group of patients.

Further, as a result of the recruitment process predominantly women with active coping strategies and an optimistic attitude applied to take part in the study. Thirdly, the themes that emerged were facilitated by means of predetermined questions, and participants would have continued conversations in three areas. This may cause some limitations to the possible themes and topics of endometriosis discussed (e.g. symptoms, medical and surgical experiences).

Finally, coping strategies and PTG in endometriosis would have been identified by using appropriate questionnaires.

## Conclusions

Uncertainty and lack of information about endometriosis as main challenges and difficulties have a significant impact on women’s life. The present findings indicate that cooperation between health care professionals, psychologists, and support organizations will be necessary for the future to provide care and find possible solutions to the needs of women living with endometriosis. Communication must be improved, and psychosocial problems need to be recognized by health care providers to ensure that empathetic care is provided. Having evidence-based answers about the efficiency of alternative and complementary therapies could decrease the uncertainty and lack of information. Furthermore, in order to reduce diagnostic delay, health care providers’ knowledge and society’s awareness of endometriosis should be improved in the near future. Health promotion programs and support groups should be managed to facilitate coping and posttraumatic growth in women with endometriosis. Achieving these recommendations would allow women to live an asymptomatic, fertile, and balanced life with endometriosis.

## Data Availability

The datasets used and/or analyzed during the current study are available from the corresponding author on reasonable request.

## Abbreviations

DIE: Infiltrating endometriosis
HRQoL: Health-related quality of life
IVF: In vitro fertilization
PTG: Posttraumatic growth
